# Comparison of Coronary Angiographic‐Derived and Intracoronary Imaging‐Based Fractional Flow Reserve in Assessing Coronary Artery Stenosis Severity: A Systematic Review and Network Meta‐Analysis

**DOI:** 10.1111/jebm.70054

**Published:** 2025-08-14

**Authors:** Jianchang Xie, Lu Ye, Jianmin Yang, Yigang Zhong, Peng Xu, Beibei Gao, Ningfu Wang, Xianhua Ye, Guoxin Tong, Jinyu Huang

**Affiliations:** ^1^ Department of Cardiology Affiliated Hangzhou First People's Hospital Westlake University School of Medicine Hangzhou China; ^2^ The Fourth School of Clinical Medicine Zhejiang Chinese Medical University Hangzhou China

**Keywords:** coronary angiography, fractional flow reserve, intravascular ultrasound, network meta‐analysis, optical coherence tomography

## Abstract

**Objective:**

This systematic review and network meta‐analysis aimed to compare the accuracy of coronary angiographic‐derived fractional flow reserve (Angio‐FFR), optical coherence tomography (OCT)‐FFR, and intravascular ultrasound (IVUS)‐FFR in evaluating the severity of coronary artery stenosis.

**Methods:**

PubMed, Embase, and Cochrane Library were searched from January 1, 2010 to April 1, 2024 for studies on the accuracy assessment of Angio‐FFR, OCT‐FFR, and IVUS‐FFR. A network meta‐analysis was performed with accuracy and analysis of variance models. The diagnostic performance was evaluated through absolute sensitivity (SEN), specificity (SPE), diagnostic dominance index (DDI), and diagnostic odds ratio (DOR), with the corresponding 95% confidence interval (CI).

**Results:**

The analysis included 86 studies (16,552 lesions). Network meta‐analysis showed that IVUS‐FFR demonstrated the highest absolute SEN of 0.92 (0.91, 0.94), while OCT‐FFR demonstrated the highest absolute SPE of 0.92 (0.91, 0.94). In comparison to Angio‐FFR, intracoronary imaging (ICI)‐FFR demonstrated superior diagnostic performance, with a DDI and DOR of 1.00 (95% CI: 0.99, 1.01) versus 0.96 (95% CI: 0.94, 0.98) and 79.18 (95% CI: 62.20, 92.35) versus 56.15 (95% CI: 52.86, 59.29), respectively. Furthermore, ICI‐FFR demonstrated significantly greater overall accuracy than Angio‐FFR, with a relative risk of 1.03 (95% CI: 1.01–1.04).

**Conclusion:**

This comprehensive network meta‐analysis establishes that ICI‐FFR provides superior diagnostic performance for coronary artery stenosis assessment compared to Angio‐FFR. These findings support the clinical value of ICI modalities in functional stenosis evaluation.

## 1 Introduction

Accumulating evidence indicates that the role of functional coronary assessment in coronary interventions is widely accepted and recommended by guidelines, particularly in the assessment of moderate lesions [1]. However, the clinical application of pressure wire‐based fractional flow reserve (FFR) is limited due to its high economic cost, prolonged surgery duration, and adenosine‐associated adverse reactions [2]. In recent years, coronary angiographic‐based functional assessment that is inferred via the fluid‐mechanics model and the flow velocity of contrast agents has been developed rapidly. Compared with pressure wire‐based FFR, coronary angiographic‐derived FFR (Angio‐FFR) exhibits excellent accuracy and rapid evaluation and is independent of pressure wire and adenosine, suggesting its promising application prospect [3].

There is an inconsistency between Angio‐FFR and pressure wire‐based FFR. The inaccurate evaluation may be associated with poor imaging, distorted or overlapped vessel development, and contrast agent underfilling [4]. Moreover, standalone FFR has inherent limitations in evaluating left main disease and vulnerable plaques. IVUS and OCT enable high‐resolution anatomical characterization of coronary structures (vessel dimensions, plaque morphology), facilitating procedural planning for stent sizing and landing zone selection [5]. When integrated with FFR, these techniques provide complementary physiological assessment to optimize interventions [6, 7]. Emerging ICI‐based FFR technologies demonstrate enhanced diagnostic performance in validation studies [6, 7], though comparative efficacy against conventional methods requires further network meta‐analysis [8].

Network meta‐analysis (NMA) refers to indirect comparisons of more than two interventions (primarily adjusted indirect comparison) or pooled meta‐analysis for direct comparisons and indirect comparisons (fixed therapeutic effects). It ranks the effect of different interventions and estimates the probability of the optimal intervention [9, 10]. There are few head‐to‐head comparisons of these FFR‐evaluating methods. It remains unclear whether a functional assessment, derived from a vessel model based on higher resolution and more practical ICI is superior to an Angio‐FFR‐derived functional assessment. This diagnostic NMA aims to compare the diagnostic performance of Angio‐FFR and ICI‐FFR with pressure wire‐based FFR as the reference standard.

## 2 Methods

### 2.1 Search Strategies and Research Sources

The NMA is conducted under the *Preferred Reporting Items for Systematic Reviews and Meta‐Analyse*s (PRISMA) statement, and the study protocol has been registered on PROSPERO (registration No. CRD42022336977) [11].

We searched PubMed, Embase, and Cochrane Library from January 1, 2010 to April 1, 2024 for relevant studies published. Core terms used to build the search strategy include “intravascular ultrasound,” “optical coherence tomography,” “coronary angiography,” “fractional flow reserve,” etc. Search strategy included a combination of free words and Medical Subject Headings (provided in the Supplementary Material).

### 2.2 Inclusion and Exclusion Criteria

Eligible studies were selected by two reviewers independently. Disagreements were settled via discussion with a third reviewer. Inclusion criteria were as follows: the study population consisted of adults with coronary artery disease and intermediate coronary stenosis; FFR calculated was based on images of coronary angiography, OCT, or IVUS; the diagnostic accuracy metrics of Angio‐FFR, OCT‐FFR, and IVUS‐FFR were compared, taking pressure wire‐based FFR as the reference standard; the cut‐off value of FFR was set as 0.8 in coronary functional assessment, and a FFR less than 0.8 indicated abnormal coronary function. Exclusion criteria were as follows: data of interest were unavailable or unextractable; FFR was calculated via machine learning; study subject was an animal model.

### 2.3 Data Extraction and Risk of Bias Assessment

Data extraction and risk of bias assessment were conducted by two reviewers independently. Any disagreement was settled via discussion with the third reviewer. Extracted data mainly included basic information (study title, first author, study design, characteristics of participants, diagnosing methods, and sample size), diagnosis‐related information (the absolute values of true‐positive, false‐positive, true‐negative, and false‐negative in the examination results, the vascular diameter stenosis in coronary angiography, sensitivity [SEN], specificity [SPE], and accuracy), and risk of bias information.

The risk of bias was assessed using the QUADAS‐2 tool in Cochrane Review Manager [12], which contains four domains: patient selection, index test, reference standard, and flow and timing. The risk of bias was assessed in each domain, and the first three domains assessed the applicability. Methodological quality assessments were categorized into three tiers of risk stratification: low risk: all signaling questions answered “yes”; high risk:≥1 critical question answered “no”; unclear risk: insufficient reporting for judgment. Overall study risk was defined as: low: all domains low‐risk; moderate:≤2 domains high/unclear‐risk without critical flaws; high: ≥3 domains high/unclear‐risk OR critical flaw in patient selection.

### 2.4 Statistical Analysis

All statistical analyses were performed using R 4.1.3. The accuracy was assessed through Bayesian NMA using the “Gemtc” package 1.0.1. The analysis of variance (ANOVA)‐based diagnostic NMA was conducted using the “rstan” 2.21.5.

NMA assessing the accuracy: the fixed‐effect and random‐effect models were applied preliminarily for fitting. The detection rate was analyzed using the relative risk (RR) with its 95% confidence intervals (CIs), which were calculated by Markov chain Monte Carlo methods. Surface under the cumulative ranking curve (SUCRA) was calculated to rank different scoring systems. The value of SUCRA ranged from 0 to 1, and a higher value indicated higher accuracy [10].

ANOVA‐based diagnostic NMA: ANOVA model needs to load “rstan” for Bayesian calculation, which can be performed via imputing “results ” stan (model_code = model, data = datalist, chains = 2, iter = 10000, warmup = 5000, thin = 1). In this instruction, the “chains” refers to the number of Monte Carlo chains, “warmup” refers to the times of pre‐iteration, “iter” the times of iteration, and “thin” the step‐length [9]. The diagnostic performance was evaluated by calculating the absolute sensitivity (ASEN), specificity (ASPE), diagnostic dominance index (DDI), and diagnostic odds ratio (DOR), with the corresponding 95% CIs. The detailed code is presented in the Supplementary Material.

## 3 Results

### 3.1 Searching Results

**FIGURE 1:**
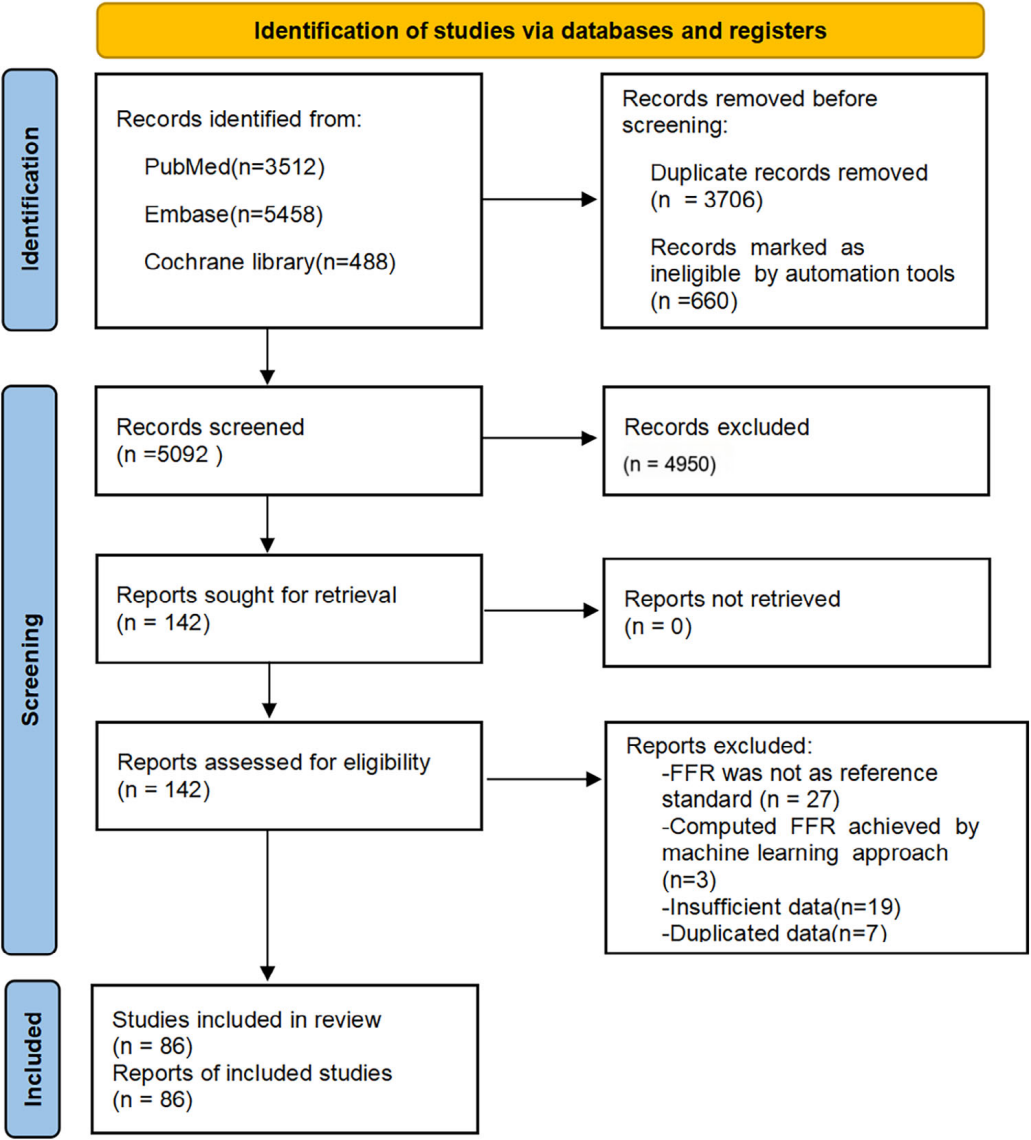
PRISMA 2020 flow diagram of search results and study attrition.

A systematic search identified 9458 potentially relevant publications. Through an automated deduplication process and preliminary quality control, 4366 records were excluded. Subsequent title/abstract screening eliminated 4950 non‐qualifying studies. The 142 potentially eligible articles underwent full‐text reviews. Following rigorous assessment, 56 studies were excluded for the following reasons: absence of wire‐based FFR as the reference standard (*n* = 27), insufficient diagnostic data (*n* = 19), duplicate datasets (*n* = 7), and FFR achieved by machine learning approach (*n* = 3). Ultimately, 86 qualified studies were included in the final quantitative synthesis. The PRISMA flow diagram of study search and selection is shown in Figure 1.

### 3.2 Characteristics of Included Studies

Among the included studies, 67 applied Angio‐FFR, 11 applied OCT‐FFR, and 8 applied IVUS‐FFR. All the studies had taken pressure wire‐based FFR as the reference standard, in which 3 studies head‐to‐head compared ICI‐FFR and Angio‐FFR [13, 14, 15] and 2 compared different kinds of Angio‐FFR [virtual FFR (vFFR), quantitative flow ratio (QFR), and FFRangio] [16, 17]. All included studies specifically assessed intermediate‐grade stenosis lesions (40%–90% diameter stenosis), with consistent inclusion criteria across studies regarding stenosis severity thresholds. The 86 included studies involved a total of 16,552 lesions, in which there were 26 prospective studies, 53 retrospective studies, 5 post hoc analyses for previous registered studies, and 2 studies with no specific description of the study design. The calculation models of QFR included fixed flow model fQFR and comparative flow model cQFR. All the related studies reported that cQFR was close to FFR, so cQFR was considered the default calculation result of QFR. Among the trials, there were 12 randomized controlled trials (RCTs), while there was 1 trial for the ICI‐FFR. Detailed characteristics of included studies are shown in Table 1.

**TABLE 1:** Characteristics of studies.

Author/study	Year	Diameter stenosis %	Number of Lesions	Index	Study design	Accuracy rate
Angiography						
Morris [18]	2013	/	35	vFFR	Retrospective single‐center	0.97
Tu [19]	2014	46.6 ± 7.3	77	FFR_QCA_	Retrospective multicenter	0.88
Tröbs [20]	2016	56 ± 14	100	FFRangio	Retrospective single‐center	0.90
Tu/FAVOR Pilot [21]	2016	64.5± 4.5	84	QFR	Prospective multicenter	0.86
Pellicano [22]	2017	/	203	FFRangio	Prospective multicenter	0.93
Emori [23]	2017	53±14(MI+)^a^ 54 ± 14(MI‐)	75 75	QFR	Retrospective single‐center	0.87 0.92
Emori [24]	2017	55 ± 10	100	QFR	Retrospective single‐center	0.94
Xu/FAVOR II China [25]	2017	46.5 ± 11.3	328	QFR	Prospective multicenter	0.93
Yazaki [26]	2017	48.8 ± 8.2	151	QFR	Retrospective single‐center	0.89
Tar [27]	2018	46	68	FFR_sim_	Retrospective multicenter	0.96
Mejía‐Rentería [28]	2018	52 ± 12	300	QFR	Retrospective multicenter	0.88
Spitaleri [29]	2018	66 ± 10	49	QFR	Retrospective multicenter	0.94
Ties [30]	2018	/	101	QFR	Retrospective single‐center	0.90
Westra/FAVOR II Europe‐Japan [31]	2018	45 ± 10	317	QFR	Prospective multicenter	0.87
Westra/WIFI II [32]	2018	50 ± 12	240	QFR	Prospective multicenter	0.83
Kornowski [33]	2018	59.8 ± 14.8	60	FFRangio	Prospective single‐center	0.95
Fearon/FAST‐FFR [34]	2019	63 ± 17	319	FFRangio	Prospective multicenter	0.92
Omori [35]	2019	64.7 ± 15.2	118	FFRangio	Prospective single‐center	0.92
Smit [36]	2019	43.2 ± 8.6	320	QFR	Retrospective single‐center	0.95
Stähli [37]	2019	41 [36–46]	516	QFR	Retrospective single‐center	0.86
Hwang [38]	2019	53.1 ± 19.0	358	QFR	Retrospective multicenter	0.93
Kleczyński [39]	2019	44.2 ± 11.7	123	QFR	Prospective single‐center	0.91
Gosling [40]	2019	58 ± 13.1	59	vFFR	Prospective single‐center	0.93
Li/FLASH FFR [41]	2020	64.2 ± 14.3	328	caFFR	Prospective multicenter	0.96
Erbay [42]	2020	41.4 [36.4–47.6] group A^b^ 41.4 [36.4–45.7] group B	516	QFR	Retrospective single‐center	0.94 0.92
Lauri [43]	2020	51.6 ± 9.8	91	QFR	Retrospective multicenter	0.84
Kanno [44]	2020	49.6 ± 12.0	504	QFR	Post hoc analysis	0.79
Mehta [45]	2020	/	85	QFR	Retrospective single‐center	0.93
Mejía‐Rentería [46]	2020	48 ± 10	138	QFR	Retrospective multicenter	0.81
Ely [47]	2020	53.3 ± 18.2	219	vFFR	Retrospective single‐center	0.68
Li [48]	2021	44 ± 12	300	AccuFFRangio	Retrospective single‐center	0.94
Ai [49]	2021	48.5 ± 9.3	170	caFFR	Retrospective single‐center	0.95
Gong/FLASH FFR [50]	2021	64.2 ± 14.3	328	cFFR	Post‐hoc analysis	0.89
Babakhani [51]	2021	/	265	NiFFR	Prospective single‐center	0.81
Choi [52]	2021	51.7 ± 16.1	599	QFR	Retrospective multicenter	0.91
Finizio [53]	2021	54 ± 8.6	100	QFR	Retrospective single‐center	0.91
Kirigaya [4]	2021	49.2 ± 8.6	160	QFR	Retrospective single‐center	0.88
Lee/C‐iFR [54]	2021	/	1077	QFR	Post‐hoc analysis	0.96
Liontou [55]	2021	51 ± 9	78	QFR	Retrospective multicenter	0.83
Milzi [56]	2021	46.1 ± 9.0	46	QFR	Retrospective single‐center	0.83
Tebaldi [57]	2021	62 [55–75]	164	QFR	Prospective single‐center	0.86
Peper [58]	2021	48.5 ± 9.4	377	QFR	Retrospective multicenter	0.88
Jin [17]	2021	44 ± 9	134	QFR	Retrospective single‐center	0.85
				vFFR		0.77
Masdjedi/FAST [59]	2021	37 ± 13	100	vFFR	Retrospective single‐center	0.86
Mileva [60]	2021	32.5 ± 16.0	39	vFFR	Prospective single‐center	0.97
Neleman/FAST EXTEND [61]	2021	/	294	vFFR	Retrospective single‐center	0.88
Tu/FAVOR II China [62]	2021	/	330	μQFR	Post‐hoc analysis	0.93
Jiang [63]	2022	45.5 ± 11.6	298	AccuFFRangio	Retrospective two centers	0.93
Wienemann [64]	2022	44.5 ± 7.5	626	QFR	Retrospective multicenter	0.94
Masdjedi/FAST II [65]	2022	42 ± 11	334	vFFR	Prospective multicenter	0.90
Aminfar [66]	2022	47 ±14	100	QFR	Prospective single‐center	0.92
Ando [67]	2022	43 ±7.4	22	AngioFFR	Retrospective single‐center	0.91
de Moura Santos [68]	2022	46.2 ± 7.4	75	QFR	Retrospective single‐center	0.84
Dowling [69]	2022	/	57	QFR	Prospective single‐center	0.84
Echavarria‐Pinto [70]	2022	46.6 ± 12.8	90	QFR	Retrospective single‐center	0.83
Kasinadhuni [71]	2022	45.25 ± 11.22	56	QFR	Retrospective single‐center	0.93
Kleczynski [72]	2022	58.6 ± 13.4	416	QFR	Prospective single‐center	0.93
Zhang [73]	2022	48.29 ± 13.2	175	QFR	Prospective multicenter	0.82
Fezzi [74]	2023	42.9 ± 16.4	381	μQFR	Retrospective single‐center	0.93
Li [75]	2023	41 ± 14	101	AccuFFRangio	Retrospective single‐center	0.96
Liang [76]	2023	/	159	AngioFFR	Retrospective single‐center	0.91
Lopez‐Palop [77]	2023	/	107	QFR	Retrospective single‐center	0.91
Omori [78]	2023	50.0 ± 12.1	253	AngioFFR	Prospective single‐center	0.88
Wang [79]	2023	44.5 ± 11.8	230	AccuFFRangio	Retrospective dual‐center	0.94
Yang [80]	2023	/	167	caFFR	Retrospective single‐center	0.91
Zuo [81]	2023	38.0 ± 8.3%	571	μQFR	Retrospective single‐center	0.87
Skalidis [16]	2024	/	84	QFR FFRangio	Prospective multicenter	0.94 0.97
OCT						
Zafar [82]	2014	45 ± 11.1	26	OCT	Retrospective single‐center	0.81
Ha [83]	2016	58.1 ± 13.4	92	OCT	Retrospective single‐center	0.88
Jang [84]	2017	56	95	OCT	Retrospective single‐center	0.86
Lee [85]	2017	/	17	OCT	Retrospective single‐center	0.94
Seike [86]	2017	55.2 ± 14.0	31	OCT	Retrospective single‐center	0.94
Yu [6]	2019	/	125	OFR	Retrospective single‐center	0.90
Emori [87]	2020	/	103	OFR	Retrospective single‐center	0.92
Gutiérrez [13]	2020	/	69	OFR	Prospective two centers	0.93
		/		QFR		0.90
Huang [14]	2020	49.4 ± 11.7	212	OFR	Retrospective single‐center	0.92
				QFR		0.87
Pan [88]	2023	48.5 ± 10.3	32	AccuFFRoct	Prospective single‐center	0.94
Jeremias/ FUSION [89]	2024	65.5 ± 14.9	266	VFR	Prospective multicenter	0.82
IVUS						
Seike [90]	2018	56.4 ± 10.7	50	IVUS	Retrospective single‐center	0.90
Bezerra [91]	2019	50 [40–60]	34	IVUS	Unknown	0.91
Siogkas [92]	2019	/	22	IVUS	Unknown	0.96
Jiang [93]	2021	/	32	AccuFFRivus	Retrospective single‐center	0.94
Yu [7]	2021	/	167	UFR	Post‐hoc analysis	0.92
Huang [94]	2023	/	47	AccuFFRivus	Retrospective single‐center	0.94
Dong [95]	2023	/	108	IVUS‐FFR	Retrospective single‐center	0.91
Yang [96]	2024	/	104	UFR	Retrospective single‐center	0.94
				QFR		0.90

*Note*: Values are mean ± SD or % or median [interquartile range].

Abbreviations: caFFR, coronary angiography‐derived fractional flow reserve; cFFR, contrast FFR fractional flow reserve; FFR_sim_, simplified computation of the FFR; FAST‐FFR, the FFRangio accuracy versus standard fractional flow reserve; FAVOR, functional assessment by various flow reconstructions; FAST, the fast assessment of stenosis severity; FUSION, validation of OCT‐based functional diagnosis of coronary stenosis; NiFFR, non‐invasive fractional flow reserve; OFR, optic flow ratio; QFR, quantitative flow ratio; QCA, quantitative coronary angiography; μQFR, Murray law‐based quantitative flow ratio; UFR, ultrasonic flow ratio; vFFR, virtual fractional flow reserve; WIFI II, wire‐free functional imaging II.

^a^ MI+, prior myocardial infarction‐related coronary arteries, MI‐, non‐prior myocardial infarction‐related coronary arteries;.

^b^ Group 1, median reference vessel diameter ≤ 2.8 mm; Group 2, median reference vessel diameter>2.8 mm.

### 3.3 Risk of Bias Assessment

**FIGURE 2:**
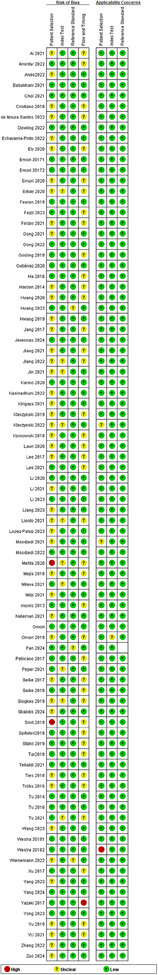
Risk of bias assessment.

The methodological quality of the included studies demonstrated a low‐to‐moderate overall risk of bias based on the QUADAS‐2 framework. Briefly, 4 studies were graded as high risk of bias, 63 studies as medium risk of bias, and 19 studies as low. The main source of risk of bias might be the few prospective studies included, specifically referring to: (1) reliance on non‐consecutive or non‐randomized patient recruitment from existing databases; (2) inadequate blinding protocols regarding wire‐based FFR results during calculated‐FFR analyses; (3) absence of real‐time FFR validation during percutaneous interventions. The results of risk of bias assessment is shown in Figure 2.

The random‐effects model analysis revealed that Angio‐FFR and ICI‐FFR exhibited moderate‐to‐high heterogeneity (*I* ^2^ = 76% and 52%). This substantial variability primarily reflects: (1) technical heterogeneity in FFR computation protocols (e.g., 11 distinct Angio‐FFR algorithms identified), (2) clinical heterogeneity in patient profiles (range of stenosis is not entirely consistent), (3) methodological limitations from retrospective designs (53/86 studies). Sensitivity analysis found a pooled DOR of 0.47 (95% CI: 0.36–0.60) and 0.51 (95% CI: 0.28–0.93), indicating robust overall diagnostic efficacy. Deeks’ tests for publication bias demonstrated a nonsignificant trend toward small‐study effects for Angio‐FFR (bias estimate = ‐7.37 ± 4.74, *p* = 0.124), and no detectable bias was observed for ICI‐FFR (bias estimate = 3.04 ± 4.78, *p* = 0.533).

### 3.4 Sensitivity and Specificity of Computational FFR

According to the ANOVA model, IVUS‐FFR had the highest ASEN of 0.92 (0.91, 0.94), and Angio‐FFR and OCT‐FFR performed similarly with the ASEN of 0.84 (0.84, 0.85) and 0.82 (0.79, 0.84), respectively. OCT‐FFR had the highest ASPE of 0.92 (0.91, 0.94) and Angio‐FFR and IVUS‐FFR also performed well with an ASPE of 0.91 (0.91, 0.92) and 0.90 (0.88, 0.92), respectively. Regarding the DDI and DOR, the IVUS‐FFR had the highest diagnostic value, with a DDI of 2.03 (1, 3) and a DOR of 126.53 (80.28, 155.45), showing a significant advantage over Angio‐FFR [DDI: 1.05 (0.33, 1), DOR: 56.49 (53.25, 59.60)] and OCT‐FFR [1.15 (0.33, 1), DOR: 61.55 (43.78, 74.14)].

Based on the overall diagnostic performance of IVUS‐FFR and OCT‐FFR, the diagnostic efficacy of ICI‐FFR was significantly better than that of Angio‐FFR, with a DDI and DOR of 1.00 (0.99, 1.01) versus 0.96 (0.94, 0.98) and 79.18 (62.20, 92.35) versus 56.15 (52.86, 59.29), respectively (Tables 2 and 3).

**TABLE 2:** AVNOVA‐based network meta‐analysis of Angio‐FFR, OCT‐FFR, and IVUS‐FFR.

	ASEN	ASPE	DDI	DOR	RSEN	RSPE
Angio‐FFR	0.84 (0.84, 0.85)	0.91 (0.91, 0.92)	1.05 (0.33, 1)	56.49 (53.25, 59.60)	0.91 (0.89, 0.93)	1.02 (1.00, 1.04)
OCT‐FFR	0.82 (0.79, 0.84)	0.92 (0.91, 0.94)	1.15 (0.33, 1)	61.55 (43.78, 74.14)	0.88 (0.85, 0.92)	1.03 (1.00, 1.06)
IVUS‐FFR	0.92 (0.91, 0.94)	0.90 (0.88, 0.92)	2.03 (1, 3)	126.53 (80.28, 155.45)	1	1

Abbreviations: ANOVA, analysis of variance; ASEN, absolute sensitivity; ASPE, absolute specificity; DDI, diagnostic dominance index; DOR, diagnostic odds ratio; FFR, fractional flow reserve; IVUS, intravascular ultrasound; OCT, optical coherence tomography; RSEN, relative sensitivity; RSPE, relative specificity.

**TABLE 3:** AVNOVA‐based network meta‐analysis of Angio‐FFR and ICI‐FFR.

	ASEN	ASPE	DDI	DOR	RSEN	RSPE
Angio‐FFR	0.84 (0.83, 0.85)	0.91 (0.91, 0.92)	0.96 (0.94, 0.98)	56.15 (52.86, 59.29)	0.96 (0.94, 0.98)	1.00 (0.99, 1.01)
ICI‐FFR	0.87 (0.86, 0.89)	0.91 (0.90, 0.93)	1.00 (0.99, 1.01)	79.18 (62.20, 92.35)	1	1

Abbreviations: ANOVA, analysis of variance; ASEN, absolute sensitivity; ASPE, absolute specificity; DDI, diagnostic dominance index; DOR, diagnostic odds ratio; FFR, fractional flow reserve; ICI, intracoronary imaging; RSEN, relative sensitivity; RSPE, relative specificity.

### 3.5 Accuracy of Computational FFR

**FIGURE 3:**
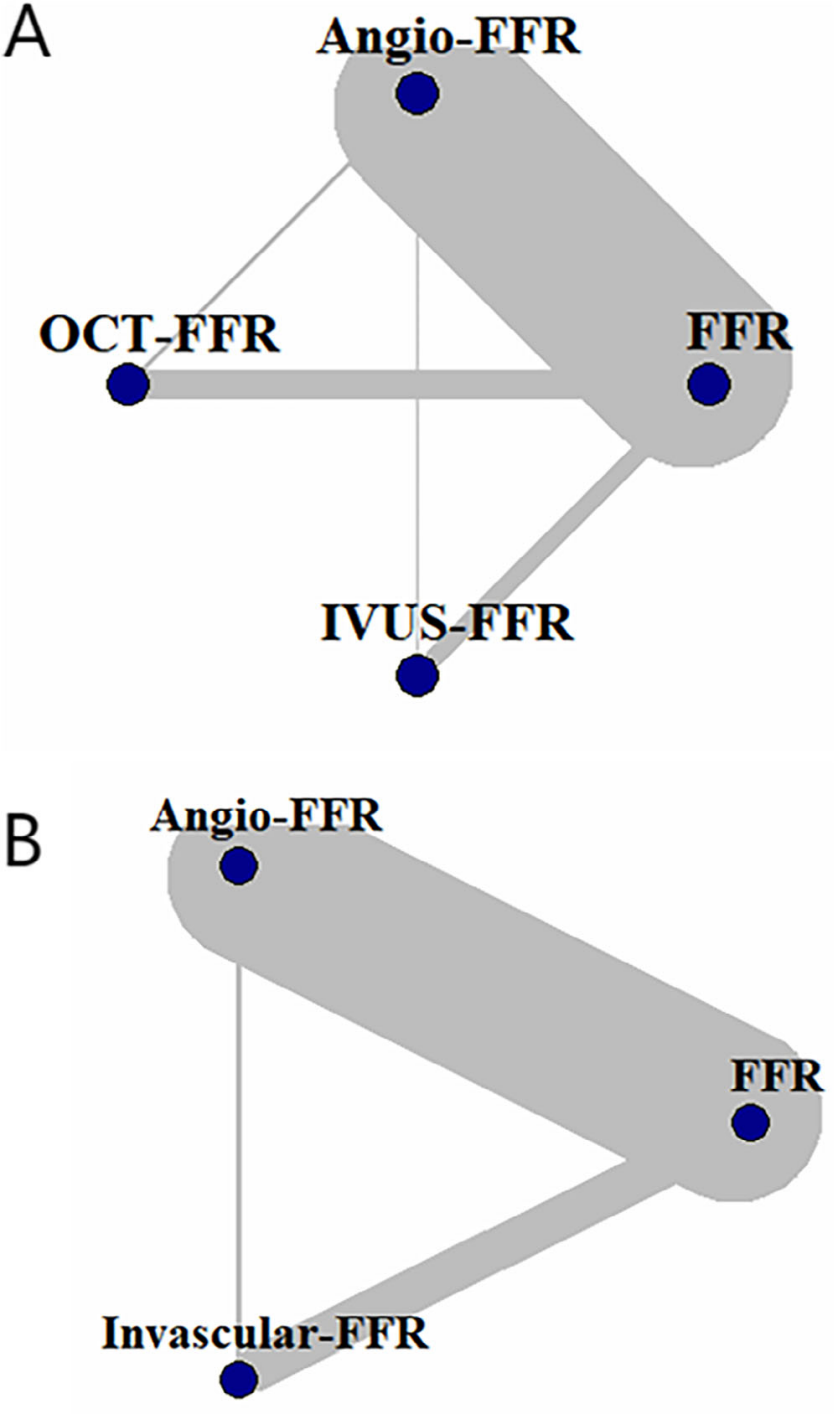
The network map demonstrates that the majority of studies have focused on separate comparisons between Angio‐FFR, IVUS‐FFR, OCT‐FFR and FFR(A), and a small number of studies have made head‐to‐head comparisons between Angio‐FFR and ICI‐FFR (B).

**FIGURE 4:**
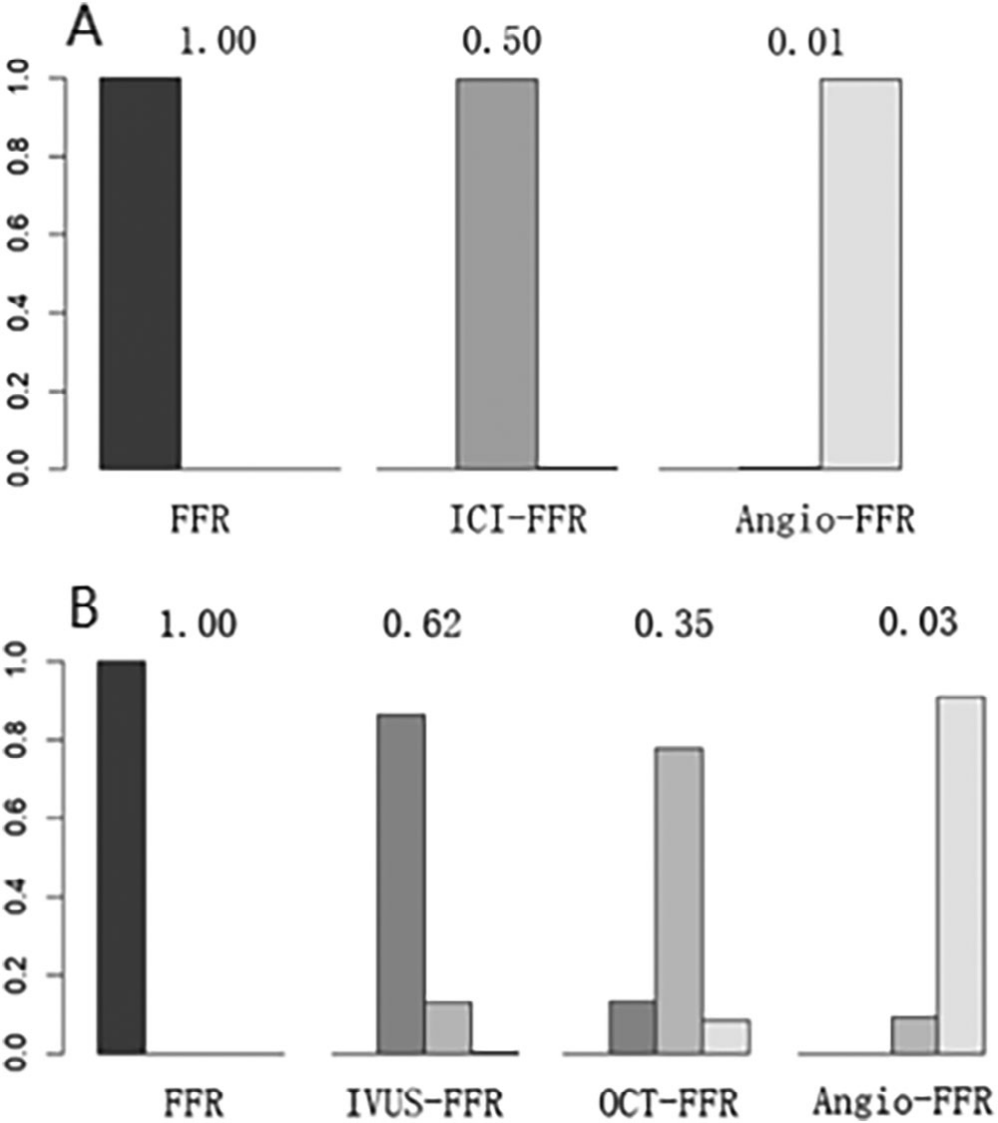
The probability histogram and the SUCRA table demonstrated that the diagnostic performance of ICI‐FFR was more closely aligned with the gold standard (FFR) than Angio‐FFR (A), while IVUS‐FFR exhibited the most optimal overall diagnostic efficacy, followed by OCT‐FFR and Angio‐FFR, respectively (B).

The three types were analyzed according to the Gemtc package. Taking FFR as the reference standard, most studies were controlled trials on Angio‐FFR and FFR, and 3 were two‐arm trials comparing OFR/UFR and QFR. The constructed network map is shown in Figure 3. Compared to Angio‐FFR, IVUS‐FFR [RR: 1.04 (1.01, 1.06)] and OCT‐FFR [RR: 1.02 (0.99 1.04)] showed better accuracy. In terms of overall performance, ICI‐FFR had a significant diagnostic accuracy advantage over Angio‐FFR (RR: 1.03, 95% CI: 1.01–1.04) (Tables 4 and 5). With FFR used as the reference standard, the probability ranking plot and the SUCRA value confirmed that IVUS‐FFR demonstrated the best performance, followed by OCT‐FFR (Figure 4B). Overall, ICI‐FFR was unequivocally superior to Angio‐FFR (Figure 4A **)**.

**TABLE 4:** League table of Angio‐FFR, OCT‐FFR, and IVUS‐FFR.

FFR			
1.11 (1.1, 1.11)	IVUS‐FFR		
1.09 (1.07, 1.11)	1.03 (1.05, 1.02)	OCT‐FFR	
1.07 (1.05, 1.01)	1.04 (1.01, 1.06)	1.02 (0.99 1.04)	Angio‐FFR

Abbreviations: FFR, fractional flow reserve; IVUS, intravascular ultrasound; OCT, optical coherence tomography.

**TABLE 5:** League table of Angio‐FFR and ICI‐FFR.

FFR		
1.1 (1.1, 1.11)	ICI‐FFR	
1.08 (1.06, 1.11)	1.03 (1.01, 1.04)	Angio‐FFR

Abbreviations: FFR, fractional flow reserve; ICI, intracoronary imaging.

## 4 Discussion

This study employed a dual‐method diagnostic network meta‐analysis to comprehensively evaluate Angio‐FFR and ICI‐FFR performance. Through the ANOVA model, we quantified both absolute metrics and direct comparative indices: IVUS‐FFR demonstrated superior absolute sensitivity (ASEN 0.92 [0.91–0.94]) while OCT‐FFR achieved optimal absolute specificity (ASPE 0.92 [0.91–0.94]); critically, this model also revealed ICI‐FFR's advantage in DDI (1.00 vs. 0.96) and DOR (79.18 vs. 56.15). Complementarily, the Bayesian NMA generated evidence‐synthesized hierarchies: a relative risk of 1.03 (95% CI: 1.01–1.04) favoring ICI‐FFR and superior SUCRA rankings confirming its diagnostic primacy. This methodological synergy—where ANOVA provides granular performance profiling but cannot integrate multi‐arm evidence, while Bayesian NMA establishes relative hierarchies through network synthesis—convergently validates ICI‐FFR's superiority for intermediate coronary stenosis assessment.

Increasing evidence supports that Angio‐FFR is the earliest‐developed and the most widely applied diagnostic method. Compared with quantitative coronary angiography (QCA), Angio‐FFR presents undoubtable merits for the diagnosis of vessel diameter stenosis [21]. The contrast agent flow model could significantly improve the diagnostic performance of QFR, compared with the fixed flow model [21, 30], which has been validated in the following FAVOR II study in China, with cQFR as the default calculation model [25]. Angio‐FFR has lower economic costs, rapid operation, the most validation studies with FFR, and continuous advantages through technological advances. For example, automated computational procedures, single angiographic view, and the introduced bifurcation fractal law are designed to improve convenience and accuracy [62, 67]. Furthermore, additional analyses for other physiological functions, such as radial wall strain and index of microvascular resistance calculation, have been developed based on coronary images [97, 98]. A FAVOR III China study revealed that QFR‐guided percutaneous coronary intervention (PCI) had significantly favorable 1‐year clinical endpoint outcomes than coronary angiography‐guided PCI [99].

Due to the inherent limitations of angiographic‐based methods, such as vascular shortening and overlap on 2D images, and interference by calcification on edge measurement, the inconsistency in functional assessment was higher in patients with acute myocardial infarction (AMI) than in patients with stable coronary heart disease [23]. This can be attributed to the early onset of microcirculation dysfunction in AMI patients. Coronary microcirculation dysfunction may compromise the diagnostic consistency between QFR and FFR [28]. On the other hand, the lack of anatomical information of the collateral vessels will affect the accuracy of the hydrodynamic model of the main collateral vessels [100]. To ensure sufficient filling of the contrast agent in the coronary artery, the angiographic catheter should be inserted into the LMCA, leading to inadequate development of the main artery. We observed that many Angio‐FFR studies usually ruled out LMCA disease.

ICI, typified by OCT and IVUS, can help further understand the structure of the vascular wall and the composition and characteristics of plaques. It is critical in guiding clinical coronary intervention and has been widely applied in the catheter room. Compared with FFR, OCT can identify high‐risk plaques like thin‐cap fibroatheroma, and this risk factor is directly related to the poor prognosis of patients, even if they have normal FFR [101, 102]. In the context of assessing ischemia‐directed PCI, FFR is a more advantageous modality, whereas ICI is better suited for assessing anatomical characteristics and planning the strategy for PCI [103]. For an intermediate coronary lesion, clinicians prefer to use FFR to assess the need for PCI and to use ICI to confirm the optimal approach for performing PCI. In low‐risk coronary arteries, FFR‐guided PCI may not be inferior to ICI‐guided PCI. However, in intermediate‐ to high‐risk complex PCI that involves long lesions and left main lesions, ICI‐guided PCI may be the more advantageous approach [104].

While ICI‐FFR demonstrates stronger correlation with wire‐based FFR than Angio‐FFR [14, 87, 105], its clinical advantage remains moderate due to three key limitations: (1) physical constraints preventing OCT/IVUS catheter access to distal small vessels; (2) use of fixed blood flow parameters rather than patient‐specific hemodynamics; (3) technical gaps in real‐time imaging‐flow integration. Emerging solutions address these: Real‐time OCT and Angiographic Co‐Registration (ACR) now provides contrast‐flow parameters for OCT‐FFR without prolonging procedures [106], while hybrid algorithms like OCT‐μQFR integrate high‐resolution plaque/stent data with functional metrics [107]. These advances enable single‐scan comprehensive assessment for optimized treatment planning.

The dual use of ANOVA model‐based diagnostic NMA and traditional Bayesian NMA is a methodological strength of this study. The former provides results such as SEN, SPE, ASEN, ASPE, and DOR, while the latter offers a comprehensive evaluation of diagnostic accuracy. These two methods are cross‐validated, and the similarity between the two sets of analytical results further ensures the reliability of the comparison outcomes. Notwithstanding the fact that the GRADE assessment is not applicable to diagnostic NMA, given the consistent findings across the different analytic models, the presence of clinically informative confidence intervals, and the fact that the primary cause of the risk of bias was non‐consecutive recruitment, the level of evidence included in this study was generally moderate and acceptable.

This study's limitations impact clinical applicability: (1) retrospective design dominance (63/86 studies) reduces real‐world generalizability by underrepresenting complex cases; (2) algorithmic differences can lead to inconsistent diagnostic performance for a particular technique and the overall performance of the system; (3) differential lesion inclusion—systematic exclusion of left main coronary artery (LMCA) lesions in Angio‐FFR studies versus inclusion in ICI‐FFR studies—restricts guidance for high‐risk anatomies; (4) technical evolution bias from ongoing computational refinements limits result relevance to current technologies; (5) evidence gaps in head‐to‐head comparisons constrain definitive superiority conclusions; and (6) substantial heterogeneity exists in some results (Angio‐FFR and ICI‐FFR exhibited moderate‐to‐high heterogeneity with *I* ^2^ = 76% and 52%), it primarily reflects technical heterogeneity in FFR computation protocol, clinical heterogeneity in patient profiles and methodological limitations from retrospective designs. These factors collectively affect clinical translation through selection bias in retrospective cohorts, protocol inconsistencies, anatomical exclusions and technological disparities, necessitating prospective trials with standardized frameworks and comprehensive lesion inclusion.

In conclusion, this NMA establishes that ICI‐FFR demonstrates superior diagnostic accuracy over Angio‐FFR for intermediate coronary stenosis. However, high heterogeneity and retrospective design dominance limit generalizability to complex scenarios. Prospective validation with a higher level of certainty will be essential for clinical implementation in the future.

## Conflicts of Interest

The authors declare no conflicts of interest.

## Supporting information




**Supporting File**: jebm70054‐sup‐0001‐SuppMat.docx
